# Rnd3 deletion affects neuroblast behavior through the RhoA/ROCK pathway but not neural stem cells in postnatal mice subventricular zone

**DOI:** 10.3389/fcell.2025.1612177

**Published:** 2025-06-23

**Authors:** Amalia Solana-Orts, Germán Belenguer, Begoña Ballester-Lurbe, Olga Gómez, Ignacio Pérez-Roger, José Terrado, Enric Poch, Alexandra Bizy

**Affiliations:** ^1^ Department of Biomedical Sciences. School of Health Sciences, Universidad Cardenal Herrera-CEU, CEU Universities, Valencia, Spain; ^2^ Centro de Investigación Biomédica en Red sobre Enfermedades Neurodegenerativas (CIBERNED), Universidad de Valencia, Burjassot, Spain; ^3^ Instituto de Biotecnología y Biomedicina (BIOTECMED), Universidad de Valencia, Burjassot, Spain; ^4^ Departamento de Biología Celular, Biología Funcional y Antropología Física, Universidad de Valencia, Burjassot, Spain; ^5^ Department of Animal Medicine and Surgery, Facultad de Veterinaria, Universidad Cardenal Herrera-CEU, CEU Universities, Valencia, Spain

**Keywords:** subventricular zone (SVZ), Rnd3/ RhoE, neural stem cell (NSC), neuroblast migration, RhoA/ROCK pathway

## Abstract

In the subventricular zone (SVZ), neural stem cells (NSCs) generate neural progenitor cells (NPCs), which proliferate and differentiate into neuroblasts (NBs) that will travel along the rostral migratory stream (RMS) to the olfactory bulbs (OBs), where they mature into interneurons. Rnd3, a member of the Rho GTPase family, regulates cytoskeletal dynamics, neuronal morphology, and survival, primarily by interacting with the RhoA/ROCK pathway. In the central nervous system, Rnd3 is highly expressed during early postnatal development and is essential for neural function, axonal myelination, and neuronal polarization, as its deficiency leads to severe motor and neurodevelopmental impairments. In this study we show that NBs from Rnd3 KO mice accumulate in the SVZ and that these are principally characterized as late/migrating NBs. We investigated whether the observed accumulation results from increased proliferation and/or differentiation potential of NSCs and NPCs, and/or altered NB migration to the OBs through the RMS, potentially accompanied by increased proliferation. Our in vitro experiments indicate that the loss of Rnd3 does not affect NSC behavior. In addition, RNA sequencing reveals that Rnd3 expression is highest in NBs, particularly in late-stage NBs, suggesting a potential role in migration. Furthermore, gene expression analyses indicate that the loss of Rnd3 may disrupt NB cytoskeletal dynamics by altering the expression of key components of the RhoA/ROCK signaling pathway. These findings provide mechanistic insights into how Rnd3 deletion impairs NB migration.

## 1 Introduction

During embryonic and early postnatal mouse development (first two to 3 weeks after birth), most neurons and glial cells in the brain originate from neural progenitors and stem cells within the neural tube (Mira and Morante, 2020). In rodents, postnatal and adult neurogenesis is primarily confined to the subventricular zone (SVZ), which lines the lateral ventricles (Fuentealba et al., 2015; Furutachi et al., 2015). Neurogenesis in the SVZ follows an indirect model, where neural stem cells (NSCs) do not generate neurons directly but instead produce neural progenitor cells (NPCs) with proliferative capacity, which undergo several rounds of division before differentiating into neuroblasts (NBs) (Haubensak et al., 2004; Miyata et al., 2004; Noctor et al., 2004). Newly formed NBs do not integrate locally but instead organize into chains and migrate along the rostral migratory stream (RMS) toward the olfactory bulb (OB), where they will mature into interneurons (Cardenas et al., 2018; Cardenas and Borrell, 2020). During early postnatal development, neuroblast production remains high, supporting the rapid growth and functional refinement of the olfactory system. In the adult brain, this process continues but at a reduced rate.

RhoE/Rnd3 (hereafter referred to as Rnd3) is a member of the Rnd subfamily of Rho GTPases, which also includes Rnd1 and Rnd2. Rho family GTPases are key regulators of actin cytoskeletal dynamics and neuronal morphology. Beyond these roles, they are also involved in neuronal survival and death and have been implicated in neurodegenerative disorders (Stankiewicz and Linseman, 2014; Basbous et al., 2021). Rnd3 belongs to the small Rnd family of atypical Rho proteins that lack intrinsic GTPase activity and remains constitutively bound to GTP (Riou et al., 2010). Rnd3 primarily functions as a negative regulator of RhoA signaling (Foster et al., 1996; Guasch et al., 1998). One of its key roles is to bind to and inhibit ROCK1, a major downstream effector of RhoA. Since ROCK1 phosphorylates LIM kinase (LIMK), which in turn phosphorylates and inactivates cofilin—a key enzyme responsible for actin filament depolymerization—Rnd3 indirectly promotes actin remodeling by maintaining cofilin activity. Additionally, Rnd3 binds to and activates p190RhoGAP, a GTPase-activating protein that further suppresses RhoA activity (Oinuma et al., 2012). Through these mechanisms, Rnd3 acts as an antagonist of RhoA, modulating the actin cytoskeleton and promoting cell motility (Pacary et al., 2011).

Beyond its role in cytoskeletal regulation, Rnd3 is involved in cell cycle control, survival, and tumor invasiveness (Hernandez-Sanchez et al., 2015). In the central nervous system (CNS), its functions have been increasingly recognized, with studies demonstrating its crucial role in neurodevelopment and neural function (Jie et al., 2015). Our laboratory has shown that Rnd3 is expressed across all CNS regions, with higher levels during early postnatal development (Ballester-Lurbe et al., 2009). In Rnd3-deficient mice, we observed severe motor and neural developmental impairments, including delayed neuromuscular synapse formation, a reduced number of motoneurons in the spinal cord, absence of the common peroneal nerve, and denervation atrophy in target muscles (Mocholi et al., 2011). Additionally, Rnd3 deficiency reduces neurite length and number, delays neuronal polarization in the hippocampus, and leads to alterations in the RhoA-ROCK-LIMK-cofilin signaling pathway (Peris et al., 2012). Recently, Rnd3 has been implicated in axonal myelination, as evidenced by significantly reduced myelin basic protein (MBP) and myelin oligodendrocyte glycoprotein (MOG) levels, as well as fewer myelinated axons and thinner myelin sheaths in Rnd3-deficient mice (Madrigal et al., 2022). In the SVZ, our previous in vivo studies demonstrated that Rnd3-deficient mice exhibit increased proliferation of cells from the SVZ and delayed neuroblast migration to the olfactory bulbs (Ballester-Lurbe et al., 2015).

Despite these findings, the role of Rnd3 in SVZ neurogenesis and NSC regulation remains poorly understood. In this study, we aimed to dissect whether Rnd3 influences the balance between different cell populations in the SVZ, specifically its impact on NSC behavior, as well as the proliferation and migration of their progeny. Our results indicate that Rnd3 deletion does not alter NSC behavior but leads to disturbed migration of NBs, specifically late-stage NBs, from the SVZ to the OBs. This impairment is likely due to altered expression of key members of the RhoA-ROCK signaling pathway, disrupting actin cytoskeleton dynamics in NBs and consequently affecting their motility. These findings highlight the crucial role of Rnd3 in NB migration, probably through the correct activation of the RhoA/ROCK pathway, providing new insights into its function in SVZ neurogenesis.

## 2 Materials and methods

### 2.1 Animal procedures

Mice were handled in accordance with European Union and national regulations. All procedures were conducted in compliance with the guidelines of the Institutional Animal Care and Use Committee (CEEA 19/004). C57BL/6NTac background wild-type and mice deficient in Rnd3 expression were derived by breeding heterozygous animals Rnd3tm1b (EUCOMM) Hmgu/H (hereafter referred to as Rnd3 KO) generated by the European Conditional Mouse Mutagenesis program (EUCOMM) using a Cre-LoxP system.

Brains from 15-days-old (P15) wild-type (WT) and Rnd3 KO mice were harvested following cervical dislocation.

### 2.2 Histological staining

The brains were fixed overnight in 4% paraformaldehyde (PFA), subsequently dehydrated through increasing concentrations of ethanol, and embedded in paraffin. Sections were cut using a microtome and mounted on polylysine-coated slides. The sections were then deparaffinized, rehydrated, and stained with a 1% cresyl violet solution.

For immunohistochemical analysis, the mouse and rabbit specific HRP/DAB (ABC) Detection IHC Kit (Abcam, Ab64264) was used. Sections were incubated with the primary antibodies overnight at 4°C in a humidified chamber. The antibodies used included NeuN (MAB377, 1:100, Millipore), PSA-NCAM (MAB5324, 1:100, Millipore). Following primary antibody incubation, the sections were treated with a biotinylated anti-rabbit or anti-mouse secondary antibody for 15 min. The detection was achieved using an HRP-labeled streptavidin complex and visualized with DAB chromogen.

### 2.3 Fluorescence-activated cell sorting (FACS)

Flow cytometry was employed to quantify cell populations from the subventricular zone (SVZ) and olfactory bulbs (OBs) following the protocol described by Belenguer et al. (2021). Brains from P15 WT and Rnd3 KO mice were extracted following cervical dislocation. The SVZ and the OBs were isolated using a dissecting microscope, and the two dissected SVZs and OBs from each brain were chopped into small pieces and transferred to a gentleMACS C tube. To obtain a single-cell suspension from the tissue, the Neural Tissue Dissociation Kit (130–093-231, Miltenyi Biotec) was used and after adding to the tissue the Enzyme Mix one and Enzyme Mix 2, the tube was placed in the gentleMACSTM tissue dissociator. Tissue pieces were mechanically dissociated and transferred to a 30 µm SmartStrainer. The resulting cell suspension was centrifuged, and the cells were stained with the appropriate antibodies. The stained cells were then analyzed using flow cytometry.

### 2.4 Establishment of a primary NSC culture

Rnd3 KO and WT SVZ were dissected under a dissecting microscope, and both SVZ from each brain were transferred into sterile, cold PBS and finely chopped into small fragments. The SVZ fragments from each brain were then transferred into a 15 mL sterile tube, and the PBS was carefully removed before proceeding to the enzymatic digestion.

One milliliter of filtered enzymatic solution (Papain 12 U/mL (Worthington biochemical), L-cistein 0.2 mg/mL (Sigma) and EDTA 0.2 mg/mL (Sigma) dissolved in EBSS (Gibco)), was pre-incubated for 30 min at 37°C and added to each brain, followed by incubation in a 37°C water bath for 30 min to facilitate tissue digestion. The tubes were then centrifuged at 1,000 rpm for 5 min, and the supernatant was carefully removed. The resulting pellet was dissociated in control medium (DMEM-F12 (Thermo Fisher), NaHCO3 0.1% (biowest), HEPES 5 mM (biowest), L-Glutamine 2 mM (Gibco), antibiotic-antimitotic (from 100x,Gibco), heparin sodium salt 0.7 U/mL (Sigma), BSA 4 mg/mL (Sigma) supplemented with hormone mix (sodium selenite 0.3 µM (Sigma), NaHCO3 0.1% (biowest), HEPES 5 mM (biowest), apo-transferrine 0.8 mg/mL (Sigma), bovine insulin 500 nM (Sigma), putrescine 0.1 mg/mL (Sigma) and progesterone 0.2 nM (Sigma) and centrifuged again. The pellet was resuspended in complete medium (control medium supplemented with the growth factors: EGF 20 ng/mL (GIBCO) and bFGF 10 ng/mL (Sigma), and individual cells were counted using trypan blue in a hemocytometer. NSCs were cultured in complete medium according to the protocols previously described (Bizy and Ferron, 2015; Belenguer et al., 2016). Concretely, for the primary culture, 20,000 cells were seeded into a 48-well plate (5 wells per brain). The plate was incubated at 37°C in a humidified incubator with 5% CO2 for 6 days.

### 2.5 Regeneration and proliferation assays

To assess NSC regeneration and proliferation, 20,000 cells were seeded in a 48-well plate. Cells were left for 6 days and the neurospheres formed in each well were counted to assess the regenerative capacity of NSCs. Images of each well were acquired to measure the diameter of the spheroids using ImageJ, to evaluate the proliferative capacity of the NSCs. Imaging was performed with an EVOS M7000 Microscope (Invitrogen). The diameters of the neurospheres were classified into the following categories: <100 μm, >100–200 μm, and >200 μm, to analyze their proliferation capacity.

### 2.6 Differentiation of NSCs

The assay was conducted using 12 mm coverslips in 24-well plates. The coverslips were sterilized and treated with Growth Factor Reduced Matrigel Matrix overnight at 37°C. Prior to cell seeding, Matrigel was removed, and the coverslips were rinsed with sterile water. A total of 100,000 cells were seeded in each well with differentiation medium I (NSC control medium supplemented with 10 ng/mL βFGF) and incubated at 37°C for 48 h. After 2 days, the medium was replaced with differentiation medium II (NSC control medium with 2% heat-inactivated, filtered FBS (0.22 µm)) and incubated at 37°C for an additional 5 days. Following the 5-day incubation, differentiation medium II was removed, and the cells were fixed with pre-warmed 2% paraformaldehyde (PFA) for 10 min at room temperature. The coverslips were then washed with PBS and stored at 4°C in PBS supplemented with 0.05% sodium azide. qRT-PCR assay was achieved at different time points of the differentiation protocol to ensure that the differentiation protocol was correctly achieved, and the differentiated cells were then quantified after immunocytochemistry.

### 2.7 Immunocytochemistry of differentiated cells

The coverslips were washed with PBS and then incubated with blocking solution (PBS with 10% FBS and 0.1% Triton X-100) for 1 h at room temperature. The primary antibody was diluted in the blocking solution and incubated for 2 h at room temperature. To stain neurons, the rabbit anti-β3-tubulin primary antibody (T2200, 1:500, Sigma-Aldrich) was used. Following primary antibody incubation, the coverslips were washed with PBS three times for 5 min each. The secondary antibody, donkey anti-rabbit (A21207, 1:800, Molecular Probes) was prepared in blocking solution and was applied for 45 min at room temperature. After washing the coverslips with PBS three times for 5 min each, they were incubated with DAPI (D9542 1:1,000, Sigma-Aldrich) for 5 min to stain the nuclei. The coverslips were then washed again with PBS and mounted with Fluorsave (345789, Sigma-Aldrich) on microscope slides. Images were captured using an EVOS M7000 Microscope (Invitrogen) and analyzed with ImageJ. Ten images were taken per animal, and the number of nuclei and neurons was counted in each image to determine the percentage of neurons relative to the total number of cells.

### 2.8 Magnetic-activating cell sorting (MACS)

To isolate NBs, we selectively dissected the SVZ and the RMS, and we followed the same protocol for enzymatic digestion as for the flow cytometry experiment. Once cell suspension was obtained, it was spun down and resuspended with anti-PSA-NCAM Microbeads (130–092-981, Miltenyi Biotec) and the PSA-NCAM positive cells were isolated through magnetic separation according to the manufacturer’s protocol.

### 2.9 Gene expression analysis

Total RNA was isolated from lysed cells using the RNeasy Micro Kit (74004, Qiagen) following the manufacturer’s protocol. The RNA concentration of each sample was measured using a Nanodrop ND-1000 spectrophotometer (Thermo Scientific) and the RNA purity was verified. 50 ng of RNA was reverse transcribed using random primers with the NZY First-Strand cDNA Synthesis kit (MB1201, NZYtech). Expression levels of different transcripts were quantified by quantitative reverse transcriptase polymerase chain reaction (qRT-qPCR) using a QuantStudio5 system (Applied Biosystems). DNA amplification was carried out with 5 ng of cDNA, TaqMan probes, and Master Mix (4304437, Applied Biosystems) according to the manufacturer’s instructions. TaqMan probes from Life Technologies were used to assess the expression of proliferation-related genes (Aspm, Cdk6, Ccnd2, and Rbbp8) and migration-associated genes (Dcx, Robo2, Enah). During the differentiation protocol, we analyzed Tubb3 to evaluate neuronal differentiation and Rnd3 to assess differences between WT and Rnd3 KO cells. Additionally, we examined the expression of RhoA/ROCK pathway components, including RhoA, ROCK, LIMK1, LIMK2, Cofilin1, and Cofilin2, to investigate potential alterations in cytoskeletal regulation. For normalization 18S ribosomal was used as housekeeping gene. Mean cycle threshold (Ct) value was first calculated as the average of duplicates for each gene of each experiment, and then dCt was calculated as each gene’s mean Ct value minus the mean Ct value of the endogenous control. Fold of expression was calculated according to the formula: fold of expression = 2 (−dCt). For PCR gene expression analysis, the expression levels of genes in the Rnd3 KO group were normalized to those in the control group. Specifically, the mean fold-change expression of the control group was set to 1, serving as the reference. Subsequently, the fold-change expression of each individual animal in the Rnd3 KO group was calculated relative to the mean fold-change expression of the control group by dividing the fold-change value for each Rnd3 KO animal by the control group’s mean fold-change expression.

### 2.10 RNA-seq data analysis

We utilized RNA-seq data previously generated and published in Belenguer et al., 2021 (GEO accession number: GSE138243). Heatmaps of normalized expression data were generated with the ComplexHeatmap R package. The top 300 genes up- or downregulated (FDR <0.05) as determined by DESeq2 with the highest positive or negative log2 fold change were used as input for Gene Ontology (GO) analysis with the ClusterProfiler R package. Single cell RNA-seq plots from P35 SVZ cells (Cebrian-Silla et al., 2021) were generated from the available dataset (https://svzneurogeniclineage.cells.ucsc.edu) deposited in the UCSC Cell Browser (Speir et al., 2021).

### 2.11 Statistical analysis

**FIGURE 1:**
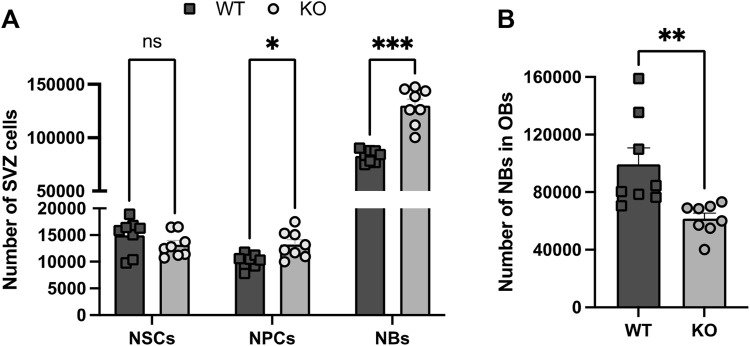
Analysis by FACS of the cellular SVZ and OB composition in WT and Rnd3 KO SVZ (N = 8 per group). **(A)** Quantification of NSCs (Lin−GLAST+CD24−CD9high), NPCs (Lin−GLAST-CD24−EGFR+ and Lin−GLAST+CD24+EGFR+) and NBs (Lin−GLAST-CD24+), and **(B)** quantification of the number of NBs in the WT and KO OBs. Data are presented as mean ± SEM. ns: not statistically significant, *p ≤ 0.05, **p ≤ 0.01, ***p ≤ 0.001 vs. WT condition. Lin−: CD45−CD31−TER119−O4−.

GraphPad Prism 10 (GraphPad Software, Inc., La Jolla, CA) was used for graph plotting and to perform statistical analyses. Quantitative data were graphed as means ± standard error of the mean (SEM). Differences between WT and Rnd3 KO samples were assessed using unpaired t-test or Mann-Whitney U test. To compare differences in various cell types between the two groups WT and Rnd3 KO (Figure 1), we performed a two-way ANOVA followed by Sidak’s multiple comparison test. Differences were considered significant when p < 0.05 for all analyses.

## 3 Results

### 3.1 Altered SVZ neurogenesis in Rnd3-deficient conditions is independent of NSC behavior

Initial findings from our laboratory obtained in a 15-day postnatal (P15) Rnd3 gt/gt mouse model, in which Rnd3 was interrupted by gene trapping, demonstrated that the mutation leads to an accumulation of NBs in the SVZ and a reduced number of neurons incorporating into the OBs (Ballester-Lurbe et al., 2015). Nevertheless, whether Rnd3 is intrinsically impairing NB proliferation and/or migration, or rather controlling NSC/NPC-derived production of NBs was not dissected. To check these possibilities, we decided to use a mouse line carrying a targeted deletion in the Rnd3 locus (Rnd3tm1b model) that results in absence of Rnd3 expression (KO mice). We first conducted a histological characterization in coronal brain sections from P15 mice (Supplementary Figure S1). Cresyl violet staining showed a significant accumulation of cells in the SVZ of Rnd3 KO mice (Supplementary Figure S1A′), leading to a broader and thicker area compared to wild-type (WT) mice (Supplementary Figure S1A). Immunostaining identified many of these supernumerary cells as immature neuroblasts (NBs), marked by PSA-NCAM positivity and NeuN negativity (Supplementary Figures S1B,B′,C,C′). As observed in sagittal sections, the accumulation was concentrated in the SVZ, with reduced presence in the RMS, indicating impaired migration of SVZ NBs to the OBs also in this genetic model (Supplementary Figures S2A,A′–C,C′). Coronal sections of Rnd3 KO OBs revealed altered size, disorganized structure, and fewer cells in the central OB region where NB chains are typically found (Supplementary Figures S2 D, D′).

Although the higher number of NBs observed in the absence of Rnd3 could be explained by a NB migration defect from the SVZ to the OBs, we next set out to explore whether an increased production of NBs could be also contributing to the Rnd3 phenotype. Because NBs are the last cell derivatives of the NSC-based neurogenic lineage, we decided to phenotype the different populations involved in the process, taking advantage of a flow cytometry-based protocol developed to phenotype the neurogenic cell population, both in the SVZ and the OB (Belenguer et al., 2021) (Figure 1).

As observed in Figure 1A, and in line with our histological analysis, we observed an increased number of NBs in Rnd3 KO vs. WT SVZs (130,001 ± 6,062 cells vs. 82,935 ± 2,026 cells respectively, p = 0.0002) while the number of NBs arriving to the OBs was significantly lower (61,632 ± 3,867 vs. 99,339 ± 11,453 NBs respectively; p = 0.0075) (Figure 1B). The cytometry-based phenotyping also indicated a very small increase of NPCs (13,240 ± 898 vs. 10,106 ± 443, p = 0.03) and no differences in the number of NSCs between WT and Rnd3 KO SVZ (Figure 1A). The data suggested that Rnd3 does not play a role in progenitor cell activity.

**FIGURE 2:**
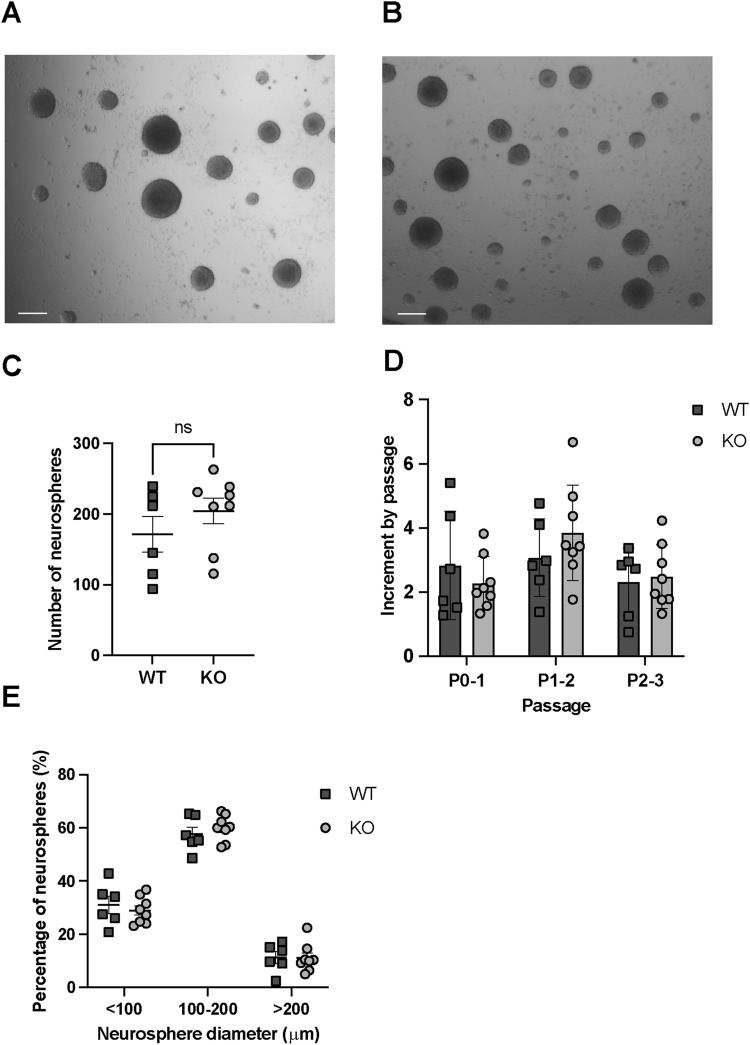
In vitro regeneration and proliferation assays in WT and Rnd3 KO NSCs (N = 6 WT and N = 8 KO). **(A)** Images of neurospheres obtained from WT and **(B)** Rnd3 KO NSCs (scale bar: 200 μm). **(C)** Number of primary neurospheres per well of a 48-well plate formed in self-renewal assays from the primary culture, **(D)** Fold increase in cell number at each passage (from P0 to P1, from P1 to P2 and from P2 to P3) in WT (N = 6) and Rnd3 KO cultures (N = 8) and **(E)** classification of neurospheres, formed 6 days after seeding from the primary culture, according to their diameter. Data are presented as mean ± SEM. ns: not statistically significant.

**FIGURE 3:**
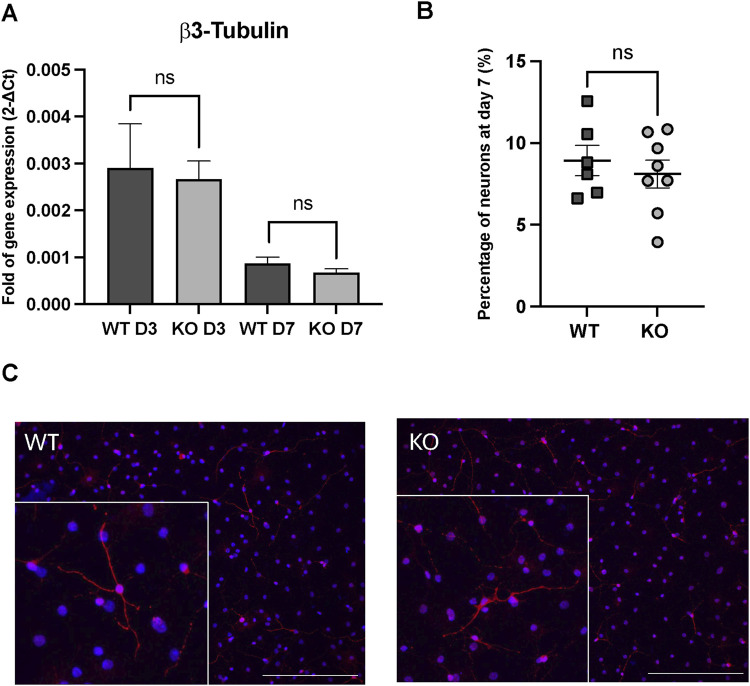
In vitro differentiation assays in WT and Rnd3 KO cultures. **(A)** Gene expression analysis of β3-tubulin through the differentiation process (at day 3 (D3) and at day 7 (D7)), **(B)** quantification of neuron percentage at day 7 of the differentiation protocol by immunofluorescence in WT (N = 6) and Rnd3 KO (N = 8) cultures and **(C)** immunofluorescence imaging of β3-tubulin as a neuronal marker (red) and DAPI for nuclei (blue). Scale bars: 200 µm. Data are presented as mean ± SEM. ns: not statistically significant.

To directly evaluate whether Rnd3 has a role in NSC behavior and/or NB production through the generation of NPCs, we decided to move to the gold-standard in vitro model of NSC function and assess the proliferative and neural differentiation capacity of Rnd3 KO NSC-derived neurospheres (Figures 2, 3). To this aim, WT and Rnd3 KO neurosphere cultures were established from P15 mice and we have conducted a qPCR analysis and did not detect Rnd3 mRNA expression in samples obtained from Rnd3 knockout neural stem cells.

First, we quantitated the number of neurospheres generated by an equal number of SVZ seeded cells from both WT (Figure 2A) and Rnd3 KO NSC cultures (Figure 2B). A comparable number of primary neurospheres (171.5 ± 25.05 in WT vs. 204.1 ± 18.01 in KO) were found in the two genotypes, in line with the cytometry data showing that NSCs are not quantitatively affected by the mutation (Figure 2C). To analyse self-renewal and growth capacity over the passages, we monitored cell proliferation from passage 0 to passage 3. Cells were counted at the end of each passage before seeding, and the fold change relative to the initial cell number was calculated. The results demonstrated similar expansion patterns between WT and KO cultures across these passages (Figure 2D). Finally, to assess proliferative capacity, we classified the neurospheres based on their diameter (Figure 2E). A comparable neurosphere size in each of the diameter ranges suggested a similar proliferative capacity of the cells involved in neurosphere formation. Together, these results demonstrate that Rnd3 is not involved in the self-renewing and proliferative capacity of NSCs.

To evaluate alterations through the progression of the neurogenic axis that could explain an increased NB production, WT and Rnd3 KO NSCs were seeded as single cells in differentiation medium following the protocol previously described (Belenguer et al., 2016). In this protocol, in vitro cultured NSCs are induced to sequentially generate proliferating NPCs and immature NBs (days 2–3) that eventually differentiate into neurons, and glial progenitors that differentiate into oligodendrocytes and astrocytes (day 7). β3-tubulin gene expression, a marker of immature neuroblasts, was analyzed at day 3 and day 7 of the differentiation process (Figure 3A). As expected, β3-tubulin gene expression diminished on day 7 because of the maturation process. However, we did not observe any difference in the expression of β3-tubulin at day 3 or at day 7 between differentiated WT and Rnd3 KO cell cultures. This result was further confirmed by β3-tubulin immunohistochemistry at day 7 (Figure 3B) showing no difference in the proportions of differentiated β3-tubulin + neurons between WT and Rnd3 KO (8.9% ± 0.93% vs. 8.1% ± 0.84, respectively, p = 0.53). We did not notice any apparent differences in neuron morphology/maturation either between genotypes (Figure 3C). Taken together, these results suggest that the deficiency of Rnd3 does not alter the neurogenic ability of NSCs/NPCs to generate new NBs or the differentiation of NBs into mature neurons.

### 3.2 Late-stage NBs acquire migrating capabilities but accumulate in the SVZ of Rnd3-deficient mice

Our previous work showed that in the absence of Rnd3, NB migration to the OBs is impaired and a higher number of BrdU + cells accumulated at the SVZ and beginning of RMS (Ballester-Lurbe et al., 2015). In this present study, we have now ruled out that NSC/NPC overproliferation or defects in cell fate transitions could play a role in the SVZ NB accumulation. Therefore, we decided to focus next on the NB population.

**FIGURE 4:**
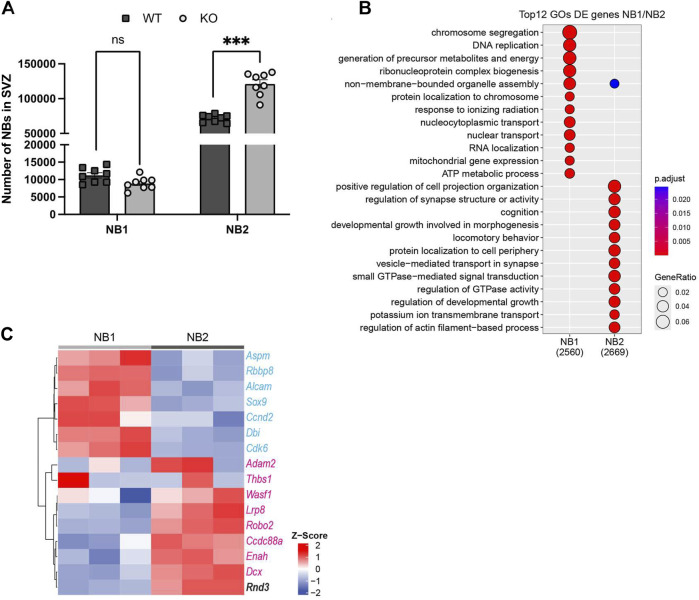
Analysis of SVZ NB transcriptomes illustrates gene expression differences associated with proliferation, migration, and Rnd3 in NB1 and NB2. **(A)** FACS quantification of early-stage NBs (Lin−GLAST-CD24+EGFR+, NB1) and late-stage NBs (Lin−GLAST-CD24+EGFR−, NB2), (N = 8 per group). **(B)** Top Gene Ontology (GO) categories of DE genes between NB1 and NB2 revealing hallmarks of proliferation and maturation and migration respectively. In brackets, the number of genes DE expressed, (N = 3 per group). **(C)** Heatmap from RNA-seq illustrating proliferation (in blue) and migration (in red) gene expression levels in NB1 (N = 3) and NB2 (N = 3). The horizontal axis identifies the cell types, distinguishing between NB1 and NB2, while the vertical axis lists the genes related to proliferation, migration, and Rnd3 expression. The color intensity on the heatmap correlates with the level of gene expression; darker colors represent higher expression levels, as detailed in the expression scale. Lin−: CD45−CD31−TER119-O4-.

The proper incorporation of new neurons to the OB requires that SVZ NB progressively cease proliferation while acquiring migrating capabilities before terminal differentiation (Ponti et al., 2013). The cytometry-based phenotyping approach indicates that early NBs (NB1) can be characterized by the expression of EGFR (epidermal growth factor receptor), the maintenance of a proliferative capacity, functionally defined through both EdU incorporation and cell cycle profiling, and a less differentiated transcriptome. In contrast, late NBs (NB2), lacking expression of EGFR, exit the cell cycle and present a more differentiated transcriptome (Belenguer et al., 2021). Moreover, pulse and chase tracing experiments have revealed that EdU+-proliferating cells of the SVZ eventually accumulate in the EGFR- NB fraction of the SVZ confirming their late stage in the neurogenic hierarchy (Belenguer et al., 2021). Based on the expression of EGFR, we further classified SVZ NBs into NB1 and NB2. Quantification of these early and late NB subtypes showed that Rnd3 KO mice SVZ exhibited a higher number of late NBs (121,116 ± 5,835 vs. 71,688 ± 1,881 NB2; p = 0.0001) compared to WT, but there was no difference in the number of early NBs (8,892 ± 635 vs. 11,236 ± 751 NB1; p = 0.84) (Figure 4A). Thus, most NBs retained in the Rnd3 KO SVZ are late-stage NBs. These results indicate that Rnd3 likely plays a role in the migration of postmitotic NBs rather than in their production from neural progenitors.

Interestingly, the analysis of available RNA sequencing (RNA-seq) data from isolated WT SVZ neurogenic cell types (Belenguer et al., 2021) shows higher Rnd3 expression in NBs, more particularly in late-stage NB2, compared to NSCs, and NPCs (Supplementary Figure S3). To further strengthen our case, we analysed an additional SVZ dataset from P30–35 mice (Cebrian-Silla et al., 2021), which is temporally closer to our experimental model. As shown in Supplementary Figure S4, Rnd3 expression strongly correlates with late stage neuroblasts (A cells with the highest pseudotime score) and with the transition from proliferation to the activation of gene ontology categories such as “neuron migration” and “synaptic transmission.” All these data support the idea of a prominent role of Rnd3 as intrinsic regulator of NB once transited from the early NB1 to the late NB2 state.

To gain insight into these populations, we then analyzed the differential expression between early and late NBs finding 2,560 genes associated to the early state and 2,669 associated to the late state. We found that top differentially expressed (DE) genes of early NBs corresponded to chromosome segregation, DNA replication, ribonucleoprotein complex biogenesis or ATP metabolic process gene ontology (GO) categories (Figure 4B). In contrast, late NBs expressed genes associated with cell projection, synapse structure, morphogenesis and locomotive behaviour or regulation of actin filament-based process GO categories, among others (Figure 4B). Importantly, late-stage NBs expressed genes implicated in small GTPase-mediated signal transduction and regulation of GTPase activity. These findings suggest potential modulation of the RhoA/ROCK signalling pathway in these cells, likely influencing cellular dynamics and potentially guiding the maturation process of late-stage NBs. In line, proliferation and migration-related genes previously reported to be relevant in NB dynamics (Khodosevich et al., 2013) clustered perfectly with early and late NBs respectively (Figure 4C). Together this analysis confirms that early EGFR + NBs correspond to an active and proliferative state and identify late EGFR- NBs as NBs acquiring migrating capabilities, and the expression of Rnd3.

Altogether, these data confirmed that the constitutive loss of Rnd3 mainly results in the accumulation of NBs in the SVZ with a migrating phenotype and a reduced number of NBs incorporating into the OBs without affecting NSCs.

### 3.3 Altered expression of migration and proliferation-related genes in Rnd3-deficient neuroblasts

Since late-stage NBs, licensed for migration, do not migrate to the OB and accumulate in the SVZ of Rnd3-deficient mice, we wondered whether the migration mechanisms of Rnd3-deficient NBs were altered. To interrogate molecular mechanisms implicated in Rnd3-associated migratory defects, we isolated NBs from both the SVZ and RMS of WT and Rnd3 KO mice using a MACS protocol, which consisted in selecting PSA-NCAM positive cells. To confirm the protocol specificity, we first verified that the fraction of PSA-NCAM-positive cells showed higher NB marker Dcx (Doublecortin) gene expression compared to the PSA-NCAM-negative cell fraction (Supplementary Figure S5).

Given the relevance of proliferation and migration-related genes in NB dynamics, we examined their expression levels in our model. This selection was based on previous findings reported in other study (Khodosevich et al., 2013) which highlighted their importance in NB behavior and that differentially clustered between early and late NBs (Figure 4C).

**FIGURE 5:**
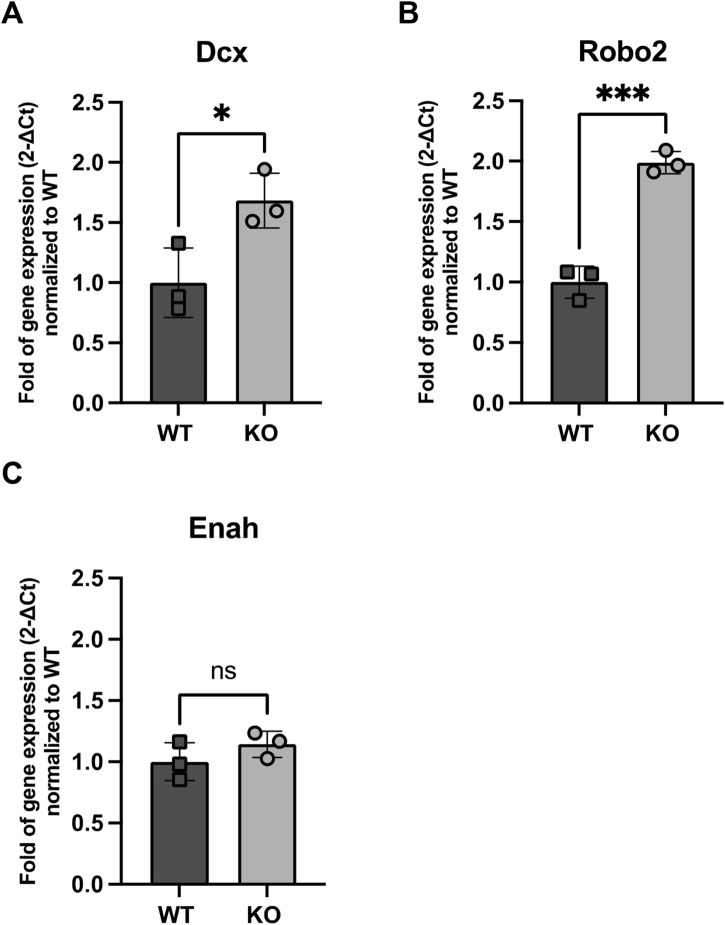
Expression of genes related to NB migration **(A)** Dcx, **(B)** Robo2 and **(C)** Enah in WT and Rnd3 KO NBs (N = 3 per group). Data are presented as mean ± SEM. ns: not statistically significant. *p ≤ 0.05, ***p ≤ 0.001 vs. WT condition.

We observed an upregulation of Dcx gene expression in KO NBs, a microtubule-associated protein critical for neuronal migration (Figure 5A), and Robo2, a receptor involved in axonal guidance (Figure 5B). Dcx gene expression was 1.68 higher in KO NBs compared to WT (p = 0.03) and Robo2 gene expression was 1.99 higher in KO NBs (p = 0.0004). In contrast, Enabled homolog (Enah), which is involved in actin polymerization and cytoskeletal reorganization, did not show a similar increase (Figure 5C).

**FIGURE 6:**
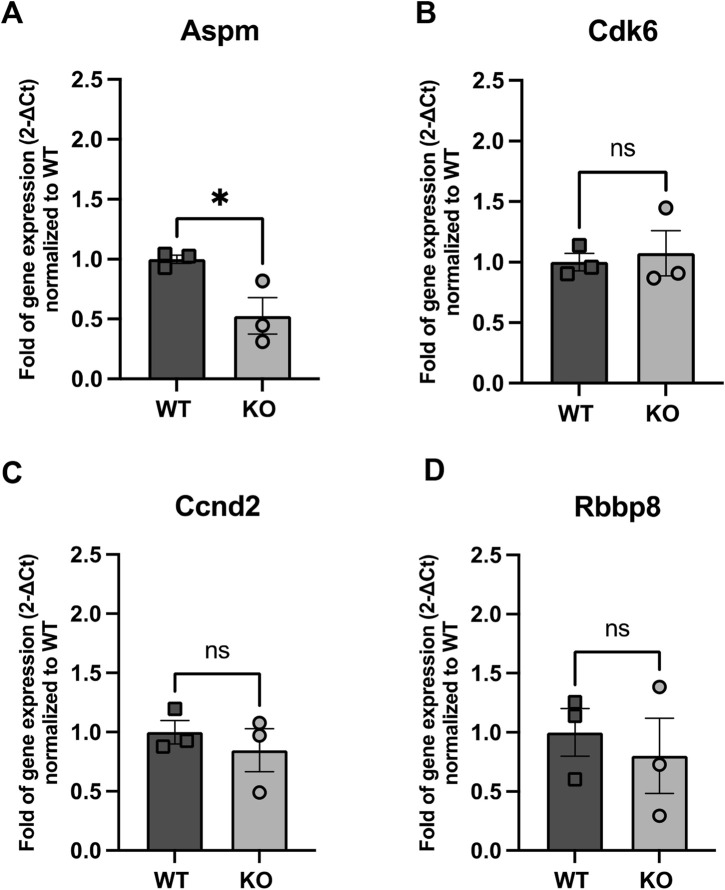
Expression of genes related to NB proliferation **(A)** Aspm, **(B)** Cdk6, **(C)** Ccnd2 and **(D)** Rbpp8 in WT and Rnd3 KO NBs. Data are presented as mean ± SEM. ns: not statistically significant. *p ≤ 0.05 vs. WT condition.

Alternatively, we also evaluated the expression of four genes implicated in cell proliferation: Abnormal spindle-like microcephaly associated (Aspm), which is crucial for normal mitotic spindle function and neuronal proliferation (Figure 6A); Cyclin-dependent kinase 6 (Cdk6), a key regulator of the cell cycle in the G1 phase (Figure 6B); Cyclin D2 (Ccnd2), which promotes cell cycle transition from G1 to S phase (Figure 6C); and Retinoblastoma-binding protein 8 (Rbbp8), which plays a role in DNA repair and cell cycle control (Figure 6D). Among these, none of them showed increased levels and even Aspm expression was significantly decreased in KO NBs (0.53-fold decrease compared to WT, p = 0.04).

Since PSA-NCAM does not differentiate between early and late NBs, we cannot exclude the possibility that this observation arises from varying proportions of early and late NBs in Rnd3KO samples. The abundance of NB2 cells in the Rnd3KO condition is consistent with higher expression of migration genes Dcx and Robo2 in these samples. On the other hand, even though the expression of Aspm is diminished in Rnd3KO NBs, the abundance of NB2 cells in the Rnd3KO condition, which either do not express proliferation genes or might express these genes excessively in the Rnd3KO condition, could mask any significant difference in overall gene expression between WT and Rnd3KO samples. The fact that the expression levels of the other three proliferation genes remained unchanged between WT and KO NBs would suggest that the transition from proliferation to migration is disrupted in the absence of Rnd3, potentially impairing the proper acquisition of a functional migration machinery in NBs.

### 3.4 Loss of Rnd3 affects the gene expression levels of the RhoA-ROCK pathway in NBs

**FIGURE 7:**
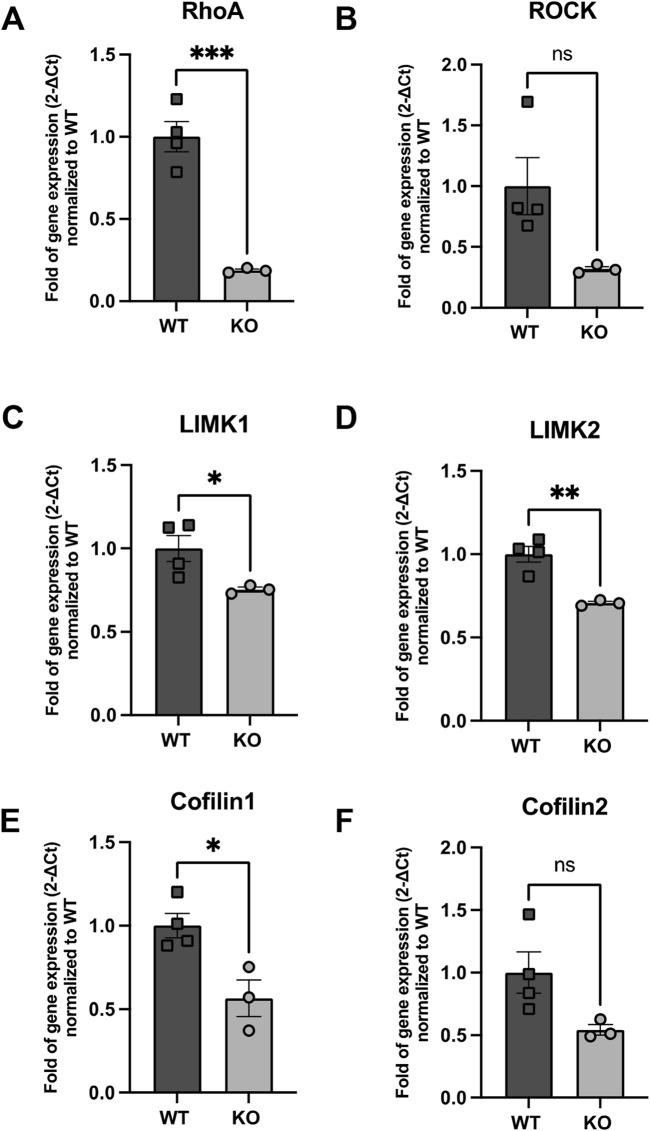
Expression of genes implicated in RhoA signaling **(A)** RhoA, **(B)** ROCK, **(C)** LIMK1 **(D)** LIMK2, **(E)** Cfl1 and **(F)** Cfl2 in WT and Rnd3 KO NBs. Data are presented as mean ± SEM. ns: not statistically significant. *p ≤ 0.05, **p ≤ 0.01, ***p ≤ 0.001 vs. WT condition.

Since NB migration is highly dependent on cytoskeletal dynamics, particularly the regulation of actin filament remodeling, we next investigated the direct impact of Rnd3 on the expression of key regulators of actin cytoskeleton dynamics (Figure 7).

Remarkably, the RNA-seq data shows that, compared to early NBs, late NBs express higher levels of ROCK1 highlighting the importance of ROCK1 for migration. In contrast, expression of Cfl1 was lower compared to early NB, indicating a predominant role for cofilin1 in proliferation (Supplementary Figure S6). This data suggests a distinct regulatory mechanism in late-stage NBs involving Rnd3 and ROCK1, where Rnd3 may be influencing the migration capabilities of late-stage NBs through modulation of ROCK1 activity. The inverse relationship observed in early-stage NBs, where Cfl1 is elevated, aligns with their proliferative nature, supporting stable actin dynamics necessary for cell division and growth. The different expression patterns of these key molecules between both NB subtypes might suggest how variations in the ROCK pathway might be integral to their distinct phenotypes, with implications for understanding how migration versus proliferation is regulated in neural development.

Indeed, the analysis of key components of actin cytoskeleton dynamics in our isolated PSA-NCAM+ cells revealed a significant downregulation of RhoA mRNA levels in Rnd3 KO NBs compared to WT (0.19-fold of gene expression normalized to WT, p = 0.0007). Similarly, we observed a reduction in the expression of Rock1, though this decrease did not reach statistical significance (p = 0.057). Furthermore, the expression levels of downstream effectors, including Limk1, Limk2 and Cfl1 were also significantly reduced in Rnd3 KO NBs compared to WT (Limk1: 0.75-fold of gene expression normalized to WT, p = 0.046, Limk2: 0.71-fold of gene expression normalized to WT, p = 0.0034, Cfl1: 0.57-fold of gene expression normalized to WT, p = 0.018) but this decrease was not significant for Cfl2 (0.54-fold of gene expression normalized to WT, p = 0.07).

Altogether, our results center the specific neurogenic role of Rnd3 in NB migration, more particularly in late-stage NBs potentially through the regulation of important players of actin-remodeling machinery and paves the road to further studies aimed at understanding the migrating mechanisms involved.

## 4 Discussion

Rnd3, an atypical member of the Rho GTPase family, plays a crucial role in cytoskeletal regulation and cell motility (Riou et al., 2010). By antagonizing RhoA-ROCK signaling, Rnd3 promotes actin cytoskeleton remodeling, facilitating NB movement along the RMS and its deletion has been associated with impaired NB migration in the SVZ (Ballester-Lurbe et al., 2015). In this work, we have deepened and answered unresolved questions regarding the role of Rnd3 in SVZ neurogenesis and NB migration, a process essential for neuronal replacement in the OBs. Understanding Rnd3’s function in this neurogenic niche could provide insights into mechanisms regulating neuroblast behavior and their implications in neurodevelopmental disorders.

### 4.1 Impact of Rnd3 on SVZ cell proliferation and differentiation

In our Rnd3 KO mouse model, natality is low, and mortality is high within the first 15 days after birth, which limits the study to postnatal development. We chose to work with P15 because, at this stage, the SVZ cytoarchitecture and cell dynamics closely resemble those of the adult brain (Sirerol-Piquer et al., 2019). Rnd3 has been identified as a key regulator of NSCs proliferation with Rnd3 deletion leading to NSC hyperplasia and an enlarged SVZ (Dong et al., 2017), but in this work we did not find any changes in NSC proliferation or differentiation in the absence of Rnd3. This discrepancy may be attributed to differences in the age of the mice; indeed, at postnatal day 3 (P3), the SVZ architecture is still in the process of maturation and differs significantly from its more structured organization at P15. At P3, the cellular composition, organization, and dynamics of NSCs are different from those observed at P15 (Tramontin et al., 2003; Sirerol-Piquer et al., 2019). It is also important to highlight that, according to our previous findings, Rnd3 expression is widely expressed in the central nervous system and that its expression decreases with age (Ballester-Lurbe et al., 2009). This could explain the difference in NSC proliferation between WT and Rnd3 KO cells at P3 but not at P15. At P15, we observed a significant accumulation of NBs in the Rnd3 KO SVZ compared to WT, yet we ruled out the possibility that this was due to altered NSC and NPC proliferation or differentiation. Interestingly, our RNA sequencing results indicate that NSCs and NPCs express very low levels of Rnd3 compared to NBs, which could explain why Rnd3 deletion does not alter NSC behavior but significantly impacts NB dynamics. However, we cannot entirely exclude the possibility that NSCs or NPCs exhibit higher proliferative and differentiative capacity at earlier stages. Furthermore, FACS analysis uncovered a markedly elevated number of NPCs in the Rnd3 KO SVZ. This finding suggests an enhanced proliferation in this population, likely driven by a niche-induced mechanism to compensate for the reduced number of NBs in the OBs.

### 4.2 Rnd3’s role in neuroblast migration and proliferation

To further investigate the impact of Rnd3 deletion on NB migration, we analyzed the expression of key migration-related genes in Rnd3 KO NBs. We observed an upregulation of Dcx, a key regulator of neuronal migration (Ayanlaja et al., 2017; Sébastien et al., 2023), and Robo2, a receptor involved in axonal guidance (Howard et al., 2021; Wurmser et al., 2021). In contrast, Enah, which plays a role in actin polymerization and cytoskeletal reorganization (Damiano-Guercio et al., 2020; Visweshwaran et al., 2022), did not show a similar increase. This suggests that the accumulation of NBs in the SVZ, unable to migrate through the RMS, may have already initiated the expression of migration-associated genes. Additionally, signals from the local niche environment could be reinforcing this upregulation through positive feedback. Also, since PSA-NCAM does not distinguish between early and late NBs, it is likely that a greater proportion of NB2 contributes to the observed increase in Dcx and Robo2 expression. The lack of Enah upregulation, despite increased Dcx and Robo2, suggests that KO NBs prioritize microtubule-based migration over actin remodeling. Since Dcx stabilizes microtubules, its upregulation may compensate for migration defects, while Enah, which regulates actin polymerization, remains unchanged due to impaired actin dynamics. If the actin cytoskeleton is already dysfunctional in KO NBs, increasing Enah expression might not be beneficial. Therefore, microtubule-based migration pathways seem to be more actively engaged, while actin-related mechanisms remain impaired due to Rnd3 loss.

NBs can undergo one to two rounds of proliferation during neurogenesis (Ponti et al., 2013). According to Belenguer et al., 2021, NBs with a profile for proliferation are classified as early-stage NBs (NB1), while those that migrate are late-stage NBs (NB2). We showed by RNA sequencing that proliferation genes are upregulated in early NBs, and migration genes in late NBs and that Rnd3 expression is higher in late NBs compared to early NBs. These findings suggest that Rnd3 is mainly important for late-stage NBs migration. To investigate if loss of Rnd3 could also alter early NB proliferation in our mouse model, we examined several key regulators of the cell cycle, including Aspm, Cdk6, Ccnd2, and Rbbp8. Only Aspm gene showed a notable reduction in expression in Rnd3 lacking NBs. We hypothesize that the selective decrease in Aspm expression may reflect a negative feedback mechanism, where the accumulation of non-migrating NBs triggers signals that suppress further proliferation, preventing excessive overcrowding in the SVZ. Rnd3 loss may selectively disrupt Aspm regulation, affecting mitotic control without altering other proliferation genes. Of note, the presence of NB2 cells in the Rnd3KO condition that either lack expression of proliferation genes or may overexpress these genes in the Rnd3KO context might attenuate differences in overall gene expression between WT and Rnd3KO samples. The unchanged expression levels of the other three proliferation genes in both WT and KO NBs indicate that the progression from proliferation to migration is disrupted without Rnd3, potentially hindering the proper development of functional migration machinery in NBs. However, we acknowledge the limitation of our analysis as we used a small number of samples (n = 3) that might make us overlook subtle, yet biologically meaningful, changes in gene expression and also, we did not separate the NB1 and NB2 cell populations; nevertheless, we are confident that our data clearly indicate that the acquisition of a migratory phenotype is impaired.

### 4.3 Mechanisms underlying migration impairments in Rnd3 KO mice

To elucidate the mechanisms through which the loss of Rnd3 impacts NB migration, we analyzed expression levels of key regulators of the actin cytoskeleton dynamics in Rnd3 KO and WT mice using quantitative RT-PCR. Our analysis revealed significant downregulation of RhoA in Rnd3 KO NBs, along with decreases in ROCK, LIMK1, LIMK2, and Cfl1 expression, while Cfl2 remained unchanged. Since the RhoA-ROCK-LIMK pathway is pivotal for actin filament stabilization and cytoskeletal remodeling, its disruption likely impairs actin turnover and motility in Rnd3 KO NBs, leading to defective migration. The role of ROCK in migration is further supported by evidence that in vivo studies, ROCK inhibition alters normal migration patterns, increasing the distribution of NPCs to ectopic brain regions (Leong et al., 2011). This suggests that while the RhoA-ROCK pathway typically supports directed cell migration, its inhibition can alter cell motility and directionality. Given that Rnd3 normally inhibits RhoA, its absence may trigger compensatory mechanisms that paradoxically reduce RhoA expression, further destabilizing the cytoskeleton and affecting cell motility. The relationship between RhoA mRNA levels and its degree of activation can be complex and depends on multiple factors, including (but not limited to) its GTP or GDP bound state (Hartmann et al., 2015). A reduced pool of RhoA protein may therefore lead to less RhoA–GTP, attenuating signaling through this pathway and potentially modulating the expression of downstream genes, including ROCK-I, to compensate for the loss of RhoA function. Also, we cannot exclude the possibility that factors beyond the expression levels of genes involved in the RhoA pathway contribute to the regulation of actin cytoskeleton remodeling in NBs.

The observed depletion of RhoA and related signaling genes in Rnd3 KO NBs correlates with impaired cytoskeletal tension and reduced cell motility, which could hinder directed NB migration. Furthermore, the downregulation of key components like ROCK, LIMK1, and LIMK2 suggests a weakened signaling cascade crucial for actin remodeling, thereby affecting NB migration efficiency. The role of ROCK signaling in maintaining structural integrity and promoting directed movement is highlighted by the significant morphological changes observed in NPCs following ROCK knockdown, including the formation of multiple protrusions and reduced migratory polarity (Vega et al., 2011). This aligns with the altered migration patterns and cellular dysfunctions seen in Rnd3 KO NBs. Additionally, RNA-seq showed that, compared to NB1, in NB2 there was high expression of ROCK1, highlighting the importance of ROCK1 for late-stage NB migration. Notably, Rnd3 levels were also elevated in NB2, suggesting that the concurrent upregulation of both Rnd3 and ROCK1 may be part of a complex compensatory mechanism. Some evidence have been recently raised about this possibility (Dai et al., 2022; Basbous et al., 2024) suggesting that even under conditions of reduced Rnd3 expression, cells can adjust ROCK1 abundance and activity to preserve cytoskeletal functions without over-stimulating the RhoA-ROCK pathway.

In contrast, expression of Cfl1 was lower in NB2 compared to NB1, indicating a predominant role for cofilin1 in proliferation. The fact that Cfl1 is not elevated when ROCK1 is high suggests that actin regulation in late-stage NBs relies on alternative pathways or compensatory mechanisms to balance cytoskeletal dynamics. This could involve post-transcriptional mechanisms, post-translational modifications like phosphorylation, feedback inhibition, or a shift in signaling priorities between migration and proliferation. This data suggests a distinct regulatory mechanism in late-stage NBs involving Rnd3 and ROCK1, where Rnd3 may be influencing the migration capabilities of late-stage NBs through modulation of ROCK1 activity. The inverse relationship observed in early-stage NBs, where Cfl1 is elevated, aligns with their proliferative nature, supporting stable actin dynamics necessary for cell division and growth. The different expression patterns of these key molecules between both subtypes suggest how variations in the ROCK pathway might be integral to the distinct phenotypes of these neuroblast subtypes, with implications for understanding how migration versus proliferation is regulated in neural development. While these intrinsic cytoskeletal defects are likely the primary cause of migration impairment, it remains possible that additional structural abnormalities in the RMS further contribute to migration impairment.

In summary, these results suggest that Rnd3 KO late-stage NBs have an intrinsic inability to migrate due to impaired actin cytoskeleton dynamics. However, in addition to this intrinsic defect, it is possible that Rnd3 KO mice also present histological abnormalities in the RMS, further hindering NB migration. Investigating potential structural defects in the RMS could provide new insights into the mechanisms underlying migration impairments, and this remains a hypothesis we aim to explore in future studies.

Since the NBs studied by MACS originate from both the RMS and SVZ, a key future perspective would be to distinguish between these two populations by separately dissecting the SVZ and RMS to compare the expression levels of proliferation and migration genes as previously done (Khodosevich et al., 2013). This approach would allow us to determine whether the observed defects come exclusively from intrinsic NB abnormalities or if additional RMS structural alterations contribute to impaired migration, a hypothesis we aim to explore in future studies. It would be interesting to check if Rnd3 KO NBs that have succeeded migrating through the RMS show a different gene expression of the Rho/ROCK pathway members than the ones that are retained in the SVZ. Additionally, future in vitro migration assays would definitely prove the migration defects we describe in this work.

A previous study led by our group on a different mouse model demonstrated that Rnd3 had an important role for the correct development of the SVZ since it affected cell proliferation, migration and differentiation and that its absence resulted in a remarkable increase of NBs proliferation in postnatal life (Ballester-Lurbe et al., 2015). The novelty of our study lies in the comprehensive analysis of Rnd3’s role in SVZ neurogenesis, particularly in its effects on NSCs and NPCs, the two different subtypes of NBs, and the RhoA/ROCK signaling pathway. We specifically examined NSC behavior, proliferation, and differentiation at P15, a stage where the SVZ cytoarchitecture and cell dynamics closely resemble those of the adult brain. Additionally, we investigated the expression of key components of the RhoA/ROCK pathway, providing mechanistic insights into how Rnd3 deletion disrupts cytoskeletal dynamics and NB migration. A main difference from the earlier study conducted on 9-day-postnatal mice is that in our study with 15-day-postnatal mice, we did not observe a NB proliferation defect since we saw neither an increased number of early-stage NBs nor increased proliferation gene expression. This is likely because, at a more adult-like stage, Rnd3’s function is primarily focused on neuroblast migration.

## 5 Conclusion

Altogether, our results center the specific neurogenic role of Rnd3 in NB migration mainly in the late-stage NBs, potentially through the regulation of important players of actin-remodeling machinery that belongs to the RhoA/ROCK pathway and paves the road to further studies aimed at understanding the migrating mechanisms involved.

## Data Availability

The datasets presented in this study can be found in online repositories. The names of the repository/repositories and accession number(s) can be found below: https://www.ncbi.nlm.nih.gov/geo/, GSE138243.

## Supplementary material

SUPPLEMENTARY FIGURE S1 Immunohistochemical labeling of coronal sections from P15 WT and Rnd3 KO mouse brains in WT **(A-C)** and Rnd3 KO **(A′-C′)** mice. **(A, A′)** cresyl violet stain, **(B, B′)** immunocytochemistry for PSA-NCAM, **(C, C′)** immunocytochemistry for NeuN. Left-side images scale bar: 1 mm; right-side images scale bar: 100 µm. Most of the cells accumulated in the Rnd3 KO SVZ are positive for PSA-NCAM, indicating they are NBs.

SUPPLEMENTARY FIGURE S2
**(A-C)** Immunohistological labeling of the SVZ sagittal sections and **(D)** OB coronal sections in P15 WT and Rnd3 KO brains. **(A, A′)** SVZ were stained with cresyl violet, **(B, B′)** immunocytochemistry for PSA-NCAM, **(C, C′)** immunocytochemistry for NeuN and **(D, D′)** OBs were stained with cresyl violet. Scale bar: 500 µm. In A′ and B′ images, arrows indicate the ventricular walls and in D and D′ images, arrows indicate the presence or absence of NBs chain in the central region of the OBs.

SUPPLEMENTARY FIGURE S3 Heatmap from RNA-sequencing illustrating Rnd3 expression levels in NSCs (N = 12), in NPCs (N = 7) and in NB1 (N = 3) and NB2 (N = 3). The color intensity on the heatmap correlates with the level of gene expression; darker colors represent higher expression levels, as detailed in the expression scale.

SUPPLEMENTARY FIGURE S4 UMAP plots from SVZ scRNAseq dataset (Cebrian-Silla et al., 2021) (top left) reveals that Rnd3 expression (top right) strongly correlates with late stage neuroblasts (A cells with the highest pseudotime score) (middle left) and with the transition from proliferation (middle right) to the activation of gene ontology categories such as “synaptic transmission” (bottom left) and “neuron migration” (bottom right).

SUPPLEMENTARY FIGURE S5 Dcx gene expression in the PSA-NCAM negative (−) fraction (N = 6) and in the PSA-NCAM positive (+) fraction (N = 6) extracted from the SVZ using the MACS technique. The PSA-NCAM + fraction isolated from the SVZ show higher Dcx expression compared to PSA-NCAM – fraction (11,5-fold of expression; p = 00018). Data are presented as mean ± SEM including individual values. **p ≤ 0.01 PSA-NCAM - fraction vs PSA-NCAM + fraction.

SUPPLEMENTARY FIGURE S6 Heatmap from RNA-sequencing illustrating Cfl1, ROCK1 and Rnd3 expression levels in NB1 and NB2. The color intensity on the heatmap correlates with the level of gene expression; darker colors represent higher expression levels, as detailed in the expression scale.
