# Supplementary figures and images for: Rnd3 deletion affects neuroblast behavior through the RhoA/ROCK pathway but not neural stem cells in postnatal mice subventricular zone
Source: Front Cell Dev Biol. 2025 Jun 23;13:1612177. doi: 10.3389/fcell.2025.1612177 (PMC12229862; doi:10.3389/fcell.2025.1612177)

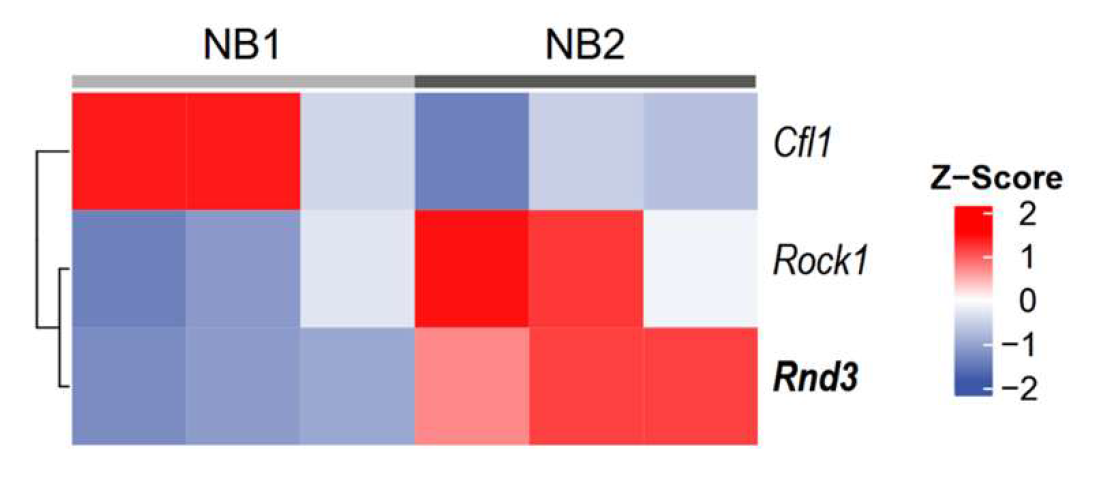

Supplement: Supplementary file 1 [file Image6.tif]

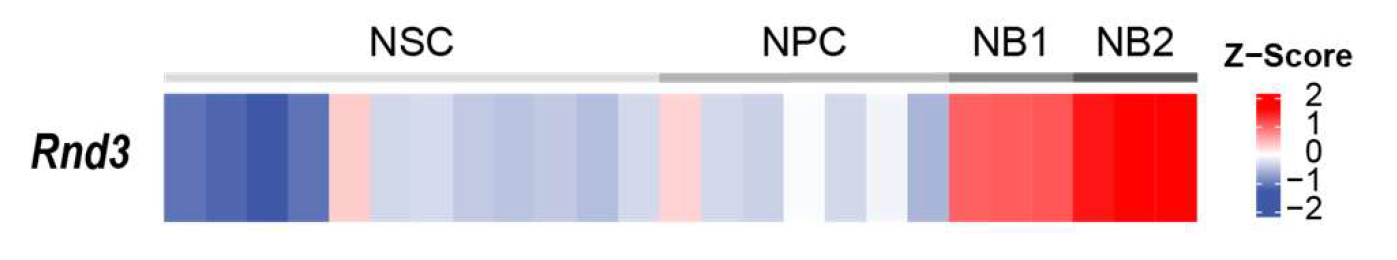

Supplement: Supplementary file 2 [file Image3.tif]

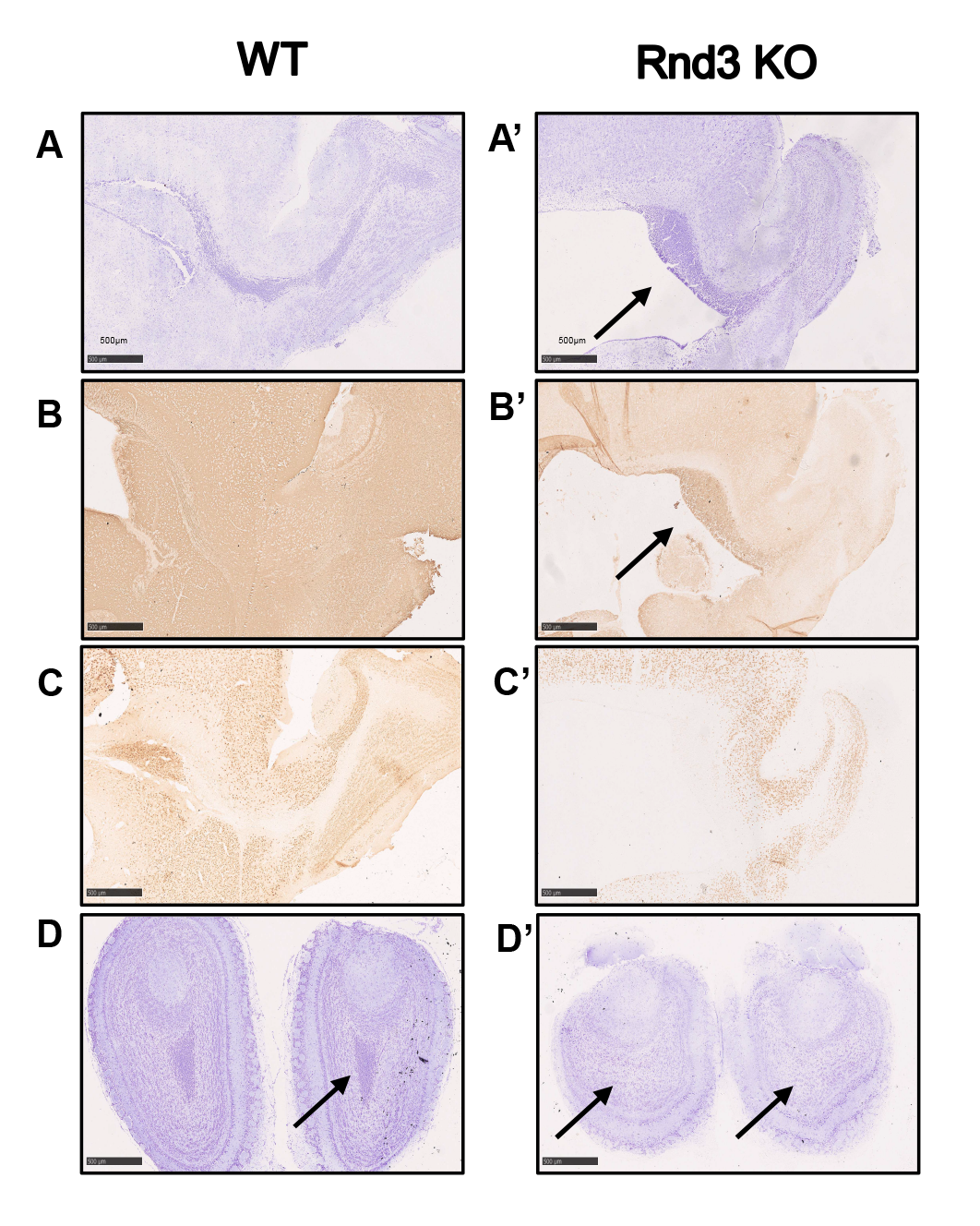

Supplement: Supplementary file 3 [file Image2.tif]

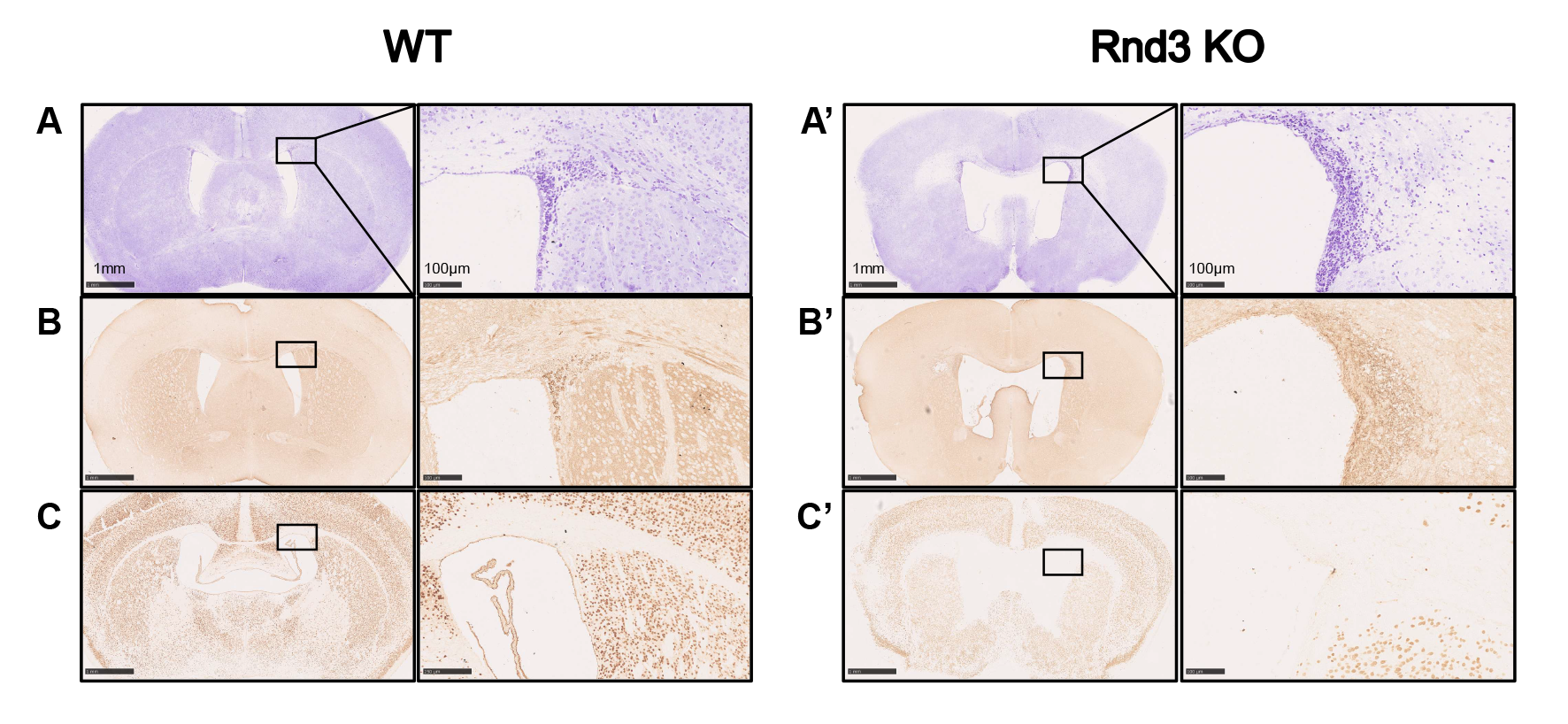

Supplement: Supplementary file 4 [file Image1.tif]

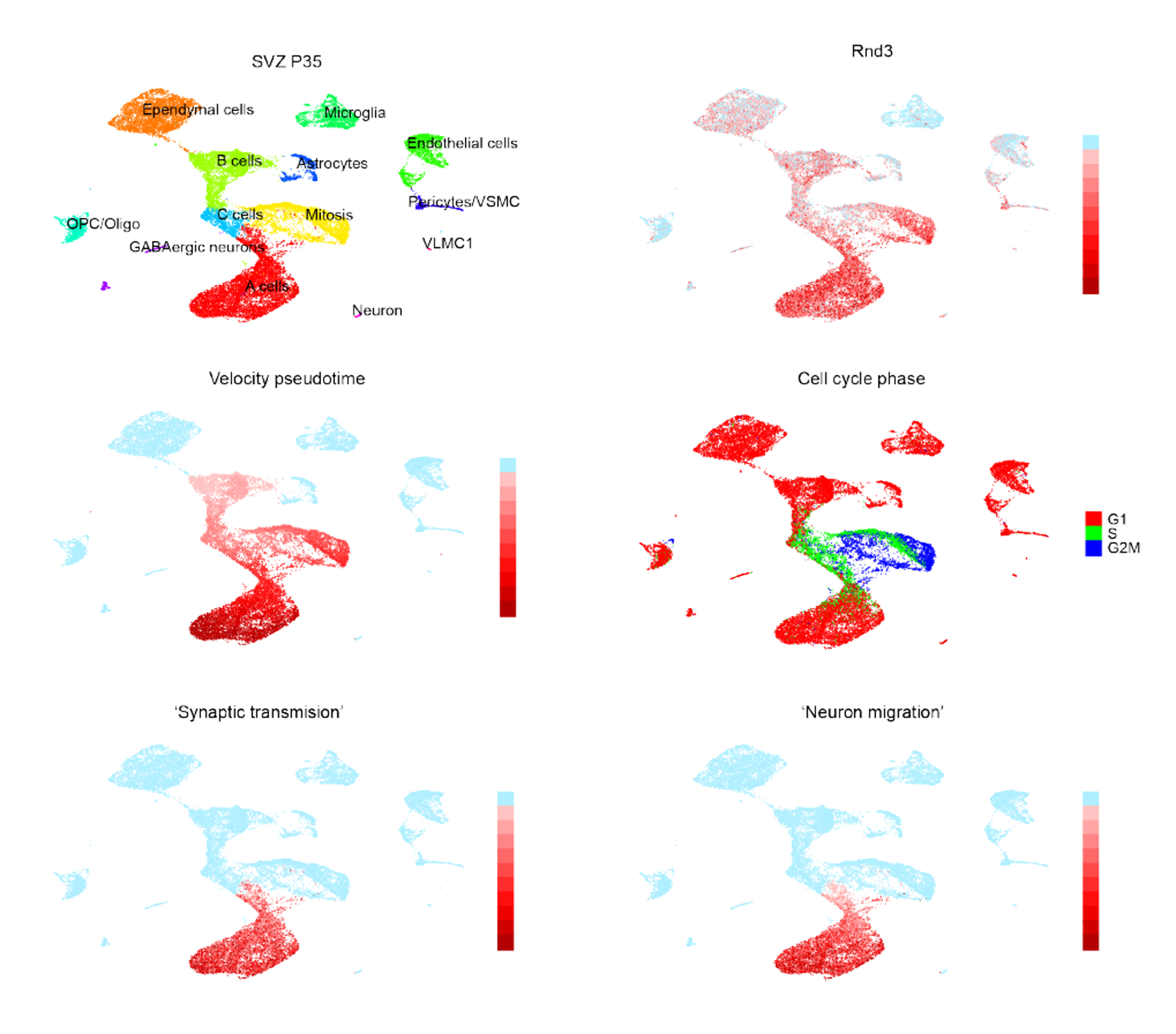

Supplement: Supplementary file 5 [file Image4.png]

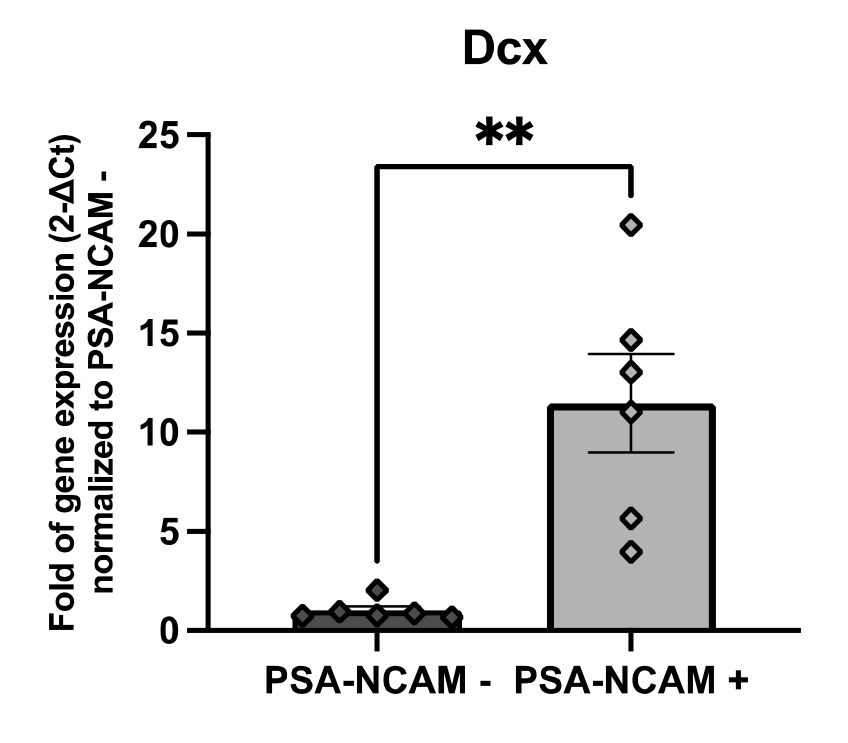

Supplement: Supplementary file 6 [file Image5.tif]
